# Aberrant promoter methylation in human DAB2 interactive protein (*hDAB2IP*) gene in gastrointestinal tumour

**DOI:** 10.1038/sj.bjc.6602458

**Published:** 2005-03-15

**Authors:** H Dote, S Toyooka, K Tsukuda, M Yano, T Ota, M Murakami, M Naito, M Toyota, A F Gazdar, N Shimizu

**Affiliations:** 1 Department of Cancer and Thoracic Surgery, Okayama University Graduate School of Medicine and Dentistry, Okayama 700-8558, Japan; 2 First Department of Internal Medicine, Cancer Research Institute, Sapporo Medical University, Sapporo 060-8543, Japan; 3 Department of Molecular Biology, Cancer Research Institute, Sapporo Medical University, Sapporo 060-8543, Japan; 4 Hamon Center for Therapeutic Oncology Research, University of Texas Southwestern Medical Center, Dallas, TX 75390, USA; 5 Department of Pathology, University of Texas Southwestern Medical Center, Dallas, TX 75390, USA

**Keywords:** methylation, hDAB2IP, gastrointestinal tumour, histone acetylation, methylation specific PCR

## Abstract

The human DOC-2/DAB2 interactive protein (*hDAB2IP*) gene is a novel member of the Ras GTPase-activating family and has been demonstrated to be a tumour-suppressor gene inactivated by methylation in several cancers. In this study, we analysed the methylation and expression status of *hDAB2IP* in gastrointestinal tumours. The promoter region of *hDAB2IP* was divided into two regions (m2a and m2b) based on our previous report, and the methylation status was determined by bisulphite DNA sequencing in gastric cancer cell lines. The gene expression was semiquantified by real-time RT–PCR, and the results indicated that the m2b promoter region might be an authentic methylation-mediated key regulator of the gene expression. Based on the sequence data, we developed a methylation-specific PCR (MSP) for the m2a and m2b regions and applied it to the samples. Methylation-specific PCR revealed aberrant methylation in the m2a region in eight of 12 gastric cancer cell lines (67%), 16 of 35 gastric cancer tissues (46%) and 29 of 60 colorectal cancer tissues (48%), and in the m2b region in eight of 12 cell lines (67%), 15 of 35 gastric cancer tissues (43%) and 28 of 60 colorectal cancer tissues (47%). On the other hand, seven (12%) and 11 (19%) of 59 gastrointestinal nonmalignant mucosal specimens showed methylation in the m2a and m2b regions, respectively, suggesting that *hDAB2IP* methylation might play a causative role in carcinogenesis. The 5-aza-2′-deoxycytidine treatment restored the gene expression in the m2b-methylated cell lines, confirming that the methylation caused gene downregulation. We also examined the relationship between *hDAB2IP* methylation and the clinicopathological features in patients with primary tumours, and determined that methylation in the m2b region was associated with location of the tumour in the stomach. In summary, our results demonstrated that *hDAB2IP* methylation is frequently present in gastrointestinal tumours and that the resulting gene silencing plays an important role in gastrointestinal carcinogenesis.

Aberrant methylation of normally unmethylated CpG islands located in the 5′ promoter region of genes has been shown to be associated with transcriptional inactivation of several genes in human cancers, including gastrointestinal tumours, and appears to serve as an alternative to mutational inactivation (Jones and Laird, 1999; Baylin and Herman, 2000).

The human DOC-2/DAB2 interactive protein gene (*hDAB2IP*), located on chromosome 9q33.1–q33.3, is a novel member of the Ras GTPase-activating family (Tseng *et al*, 1998; Chen *et al*, 2002; Wang *et al*, 2002). It interacts directly with the DOC-2/DAB2 (also known as differentially expressed in ovarian carcinoma-2, DOC-2) protein that appears to be a tumour suppressor in malignant tumours, including mammary, prostate and ovarian cancers (Fulop *et al*, 1998; Zhou and Hsieh, 2001). *hDAB2IP* and DAB2 form a unique protein complex and exert a negative regulatory activity on the Ras-mediated signal pathway (Wang *et al*, 2002). Recently, it was reported that the P2 region (−598 to +44) of the *hDAB2IP* gene shows promoter activity, and that transcriptional silencing by aberrant methylation of the P2 region is critical tumorigenesis in prostate cancer (Chen *et al*, 2003) and breast cancer (Dote *et al*, 2004).

In this study, we examined the methylation and expression status of *hDAB2IP* in a series of gastric cancer cell lines, and developed the methylation-specific PCR (MSP) assay for *hDAB2IP* for application to gastrointestinal tumour samples. To clarify the mechanism of gene silencing, the cell lines were treated with 5-aza-2′-deoxycytidine (5-Aza-CdR) and/or trichostatin A (TSA) to examine the effect of methylation and histone deacetylation on the gene silencing. In addition, we analysed the relationship between the methylation status and the clinicopathological features in cases with surgically resected gastric and colorectal cancers, in order to investigate the clinical and pathogenetic importance of *hDAB2IP* methylation in gastrointestinal tumours.

## MATERIALS AND METHODS

### Cell lines

In all, 12 cell lines derived from human gastric cancer were used in this study. Four gastric cancer cell lines of MKN series (MKN-1, adenosquamous cell carcinoma; MKN-28 and MKN-74, well-differentiated adenocarcinoma; MKN-45, poorly differentiated adenocarcinoma) and KATO-III (signet ring cell carcinoma) were kindly provided from Professor K Shimizu (Department of Molecular Genetics, Okayama University Graduate School of Medicine and Dentistry, Okayama, Japan). NCI-SNU-5 and NCI-SNU-16 (poorly differentiated adenocarcinoma) were obtained from the American Type Culture Collection (Manassaa, VA, USA). The other five cell lines (MKN-7, well-differentiated adenocarcinoma; NUGC-4, signet ring cell carcinoma; SH-10-TC, poorly differentiated adenocarcinoma; AZ521 and H-111-TC, well-differentiated adenocarcinomas) were obtained from Cell Resource Center for Biomedical Research, Tohoku University. Most cells were grown in RPMI1640 (Sigma Chemical Co., St Louis, MO, USA) supplemented with 10% heat-inactivated fetal bovine serum and incubated in 5% CO_2_.

### Clinical samples

Surgically resected specimens of 35 primary gastric cancers, 60 primary colorectal cancers and 59 matched corresponding nonmalignant mucosa specimens were obtained from Okayama University Hospital, Okayama, Japan after acquiring informed consent from each patient between 1998 and 2002. Patient demographics were shown in Table 1 . Histological classification and tumour staging were carried out according to Lauren classification (Lauren, 1991) and the TNM Stage Grouping (Sobin and Fleming, 1997). Peripheral blood lymphocytes were obtained from five healthy volunteers. The whole procedure was approved by the Appropriate Institutional Review Board of our centre.

### DNA extraction and bisulphite treatment

DNA from cell lines and specimens was subjected to bisulphite treatment as described previously (Dote *et al*, 2004). Briefly for bisulphite treatment, 1 *μ*g of genomic DNA was denatured by NaOH and modified by sodium bisulphite, which converts all unmethylated cytosines to uracils while methylated cytosines remain unchanged (Wang *et al*, 1980). The modified DNA was purified using a Wizard DNA cleanup system (Promega, Madison, WI, USA).

### Map of 5′-flanking region of *hDAB2IP* and bisulphited DNA sequencing analysis

**Figure 1:**
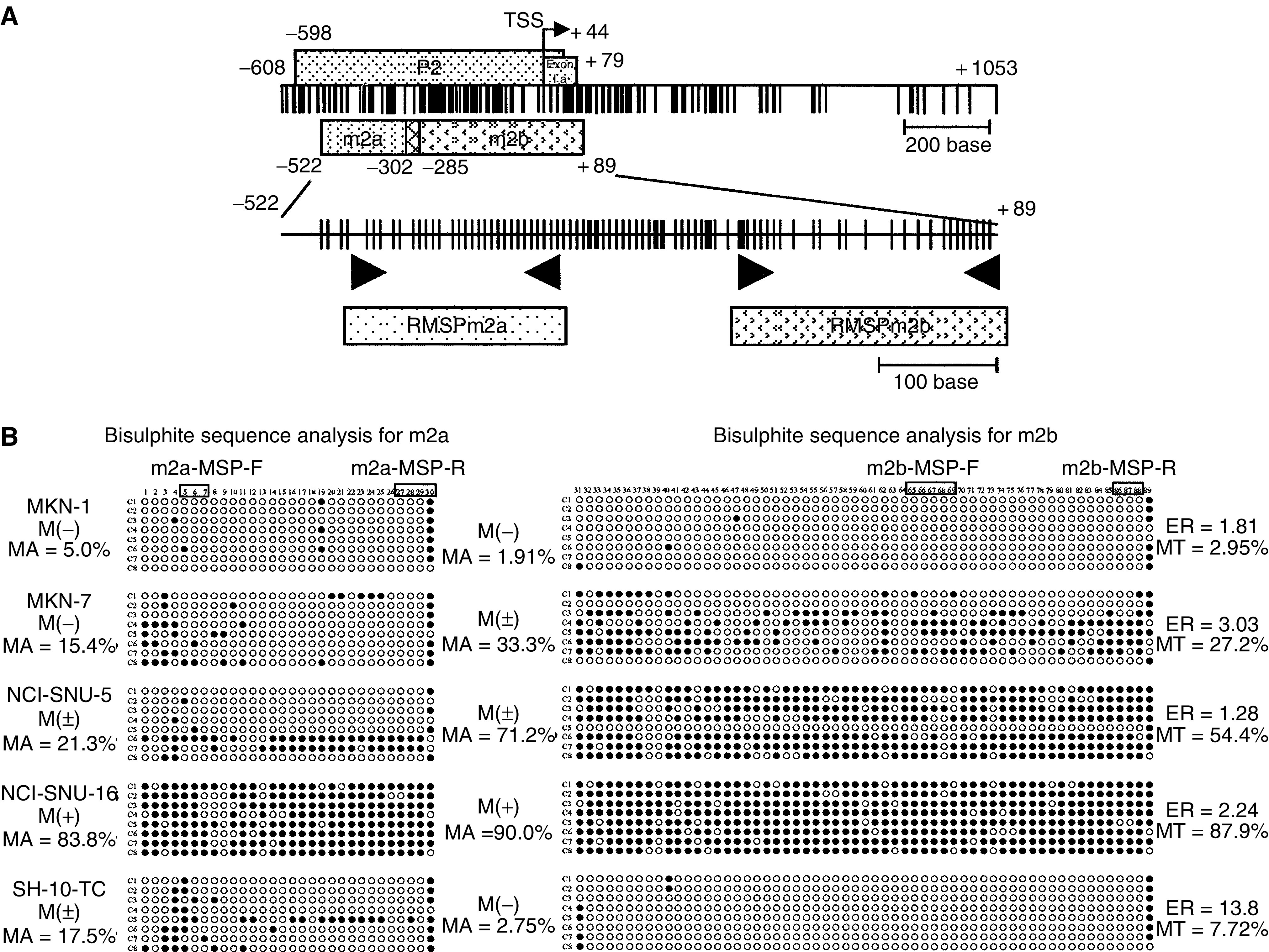
Map of 5′ flanking region of *hDAB2IP* gene and the bisulphited genomic DNA sequence. (**A**) Location of CpG dinucleotides in the genomic sequence (GenBank accession number AL365274) are indicated by *thin vertical lines*. The bent arrow indicates the transcription start site (TSS) (+1). *Five rectangular boxes*, promoter region (*P2*), regions for the bisulphited genomic sequence (*m2a*: from −522 to −285*, m2b*: from −308 to +89) and regions for the amplicons of MSP (*RMSPm2a, RMSPm2b*). (**B**) Methylation status of individual cloned DNA fragments of five gastric cancer cell lines is shown. Each *row* represents one sequenced allele. Each *circle* represents a CpG dinucleotide. *Filled circle*, methylation; *open circle*, no methylation. Clonal numbers are indicated by prefix C to the *left*. The *numbers* at the top indicate the CpG dinucleotide in the amplicon (5′ to 3′). The percentage of *MA*, *MB* and *MT* indicates the rate of methylated CpG dinucleotides in m2a, m2b and total regions of each sample, respectively. *ER* means expression ratio by semiquantitative real-time RT–PCR. The location of CpG dinucleotides included in MSP primers (m2a-MSP-F, m2a-MSP-R, m2b-MSP-F and m2b-MSP-R) are indicated by *open boxes*. *M (+)*, positive for the *hDAB2IP*-methylated form by MSP; *M (−)*, negative for the *hDAB2IP*-methylated form by MSP; *M (±)*, positive for both the *hDAB2IP*-methylated and -unmethylated forms by MSP.

Our previous report identified the promoter region of *hDAB2IP* (P2) that was divided into m2a (237 bp) and m2b (401 bp) regions (Chen *et al*, 2003). The location of the CpG dinucleotides, P2, m2a and m2b regions in the 5′-flanking region of *hDAB2IP* (GenBank accession number AL365274) are shown in Figure 1A. The m2a and m2b were amplified and PCR amplicons were cloned into pCR2.1-TOPO Vector and sequenced following our previous report (Chen *et al*, 2003). To determine the methylation status of *hDAB2IP*, individual eight clones from each sample were sequenced by the Applied Biosystems PRISM dye terminator cycle sequencing method (Perkin-Elmer Corp., Foster City, CA, USA) using forward and reverse M13 primers.

### *hDAB2IP* mRNA expression by semiquantitative real-time RT–PCR

Total cellular RNA was isolated from cell lines and five nonmalignant gastric mucosa specimens with RNeasy minikit (Qiagen Sciences, MD, USA) according to the manufacturer's instructions and then treated with 2 units *μ*l^−1^ of DNase I (Ambion, Austin, TX, USA) for 30 min at 37°C. RT reaction was performed for 2 *μ*g of total RNA with the SuperScript II First-strand Synthesis using the Oligo (dT) primer system (Invitrogen Life Technologies, Inc., Carlsbad, CA, USA) in 20 *μ*l reaction mixture. cDNAs were semiquantified by fluorescence-based real-time RT–PCR using TaqMan technology (Perkin Elmer Corp., Foster City, CA, USA) with the Gene Amp 5700 Sequence Detection System (Perkin Elmer Corp.). TATA box binding protein (*TBP*) was used to normalise the expression of *hDAB2IP* (Pawlowski *et al*, 2000; Toyooka *et al*, 2001). The sequences of the primers and probe for *hDAB2IP* and *TBP* expression were previously reported. We used serial dilutions of the positive control cDNA to create a standard curve. The expression ratio was defined as the *hDAB2IP* PCR products compared to those of the *TBP*, multiplied by 100. All experiments were performed in duplicate.

### MSP assay

The methylation-specific primers were designed based on the bisulphited DNA sequencing data of gastric, breast (Dote *et al*, 2004) and lung (data not shown) cancer cell lines, whose expressions were remarkably downregulated (see below). The location of MSP primers was shown in Figure 1A and B. The methylation status of m2a and m2b regions in gastric and colorectal cancers was determined by MSP (Herman *et al*, 1996). The methylated and unmethylated alleles for m2a and m2b regions were amplified using *hDAB2IP*m2a-MSP-F and *hDAB2IP*m2a-MSP-R for methylated m2a, *hDAB2IP*m2b-MSP-F and *hDAB2IP*m2b-MSP-R for methylated m2b, *hDAB2IP*m2a-USP-F and *hDAB2IP*m2a-USP-R for unmethylated m2a, and *hDAB2IP*m2b-USP-F and *hDAB2IP*m2b-USP-R for unmethylated m2b. The PCR products were resolved by electrophoresis in a 2% agarose gels containing ethidium bromide. The normal lymphocyte DNA that was treated with Sss1 methyltransferase (New England BioLabs, Bevely, MA, USA) and then subjected to bisulphite treatment was used as a positive control for methylated alleles. Water blanks were included in each assay.

### 5-Aza-CdR and TSA treatment

Cell lines were treated with 5-Aza-CdR (Sigma-Aldrich Co., St Louis, MO, USA) at a concentration of 1–2 *μ*g ml^−1^ for 6 days with medium changes on days 1, 3 and 5 (Jones, 1985; Virmani *et al*, 2000). The treatment with histone deacetylase inhibitor, TSA (Wako, Tokyo, Japan), was performed at a concentration of 150–300 nM for 12–24 h (Yoshida *et al*, 1990; Toyooka *et al*, 2002a). For combination treatment, TSA was added to day 5 of 5-Aza-CdR-treated cell lines and cultured for additional 12–24 h. Treated or untreated cells from individual triplicate flasks were harvested to be semiquantified by the gene expression level using real-time RT–PCR as described above.

### Statistical analysis

The frequencies of *hDAB2IP* methylation between two groups were compared using the *χ*^2^ test. The quantitative ratios of different groups were compared using the Mann–Whitney *U*-nonparametric test. Probability value less than 0.05 was defined as being statistically significant. All data were analysed with StatView for Windows (SAS Institute Inc., Cary, NC, USA).

## RESULTS

### Bisulphited genomic DNA sequencing

The m2a and m2b regions, covering the major part of the P2 region, were separately amplified by PCR to examine their methylation status by direct sequencing in eight gastric cancer cell lines, and five cell lines that showed one of the four typical methylation patterns were selected for more detailed analysis. The PCR amplicons of these five cell lines were cloned, and eight individual clones were sequenced (Figure 1B). MKN-1 showed almost no methylation in either the m2a or the m2b region. In contrast, NCI-SNU-16 showed a heavy methylation pattern, with methylation of over 80% of all the CpG dinucleotides in both the m2a and m2b regions. The other three cell lines showed a moderate methylation pattern, which was subdivided into two types, the m2a- and the m2b-dominant methylation pattern. Two cell lines, namely, MKN-7 and NCI-SNU-5, showed an m2b-dominant methylation pattern, and one cell line, namely, SH-10 TC, showed an m2a-dominant methylation pattern.

### *hDAB2IP* expression in gastric cancer cell lines

**Figure 2:**
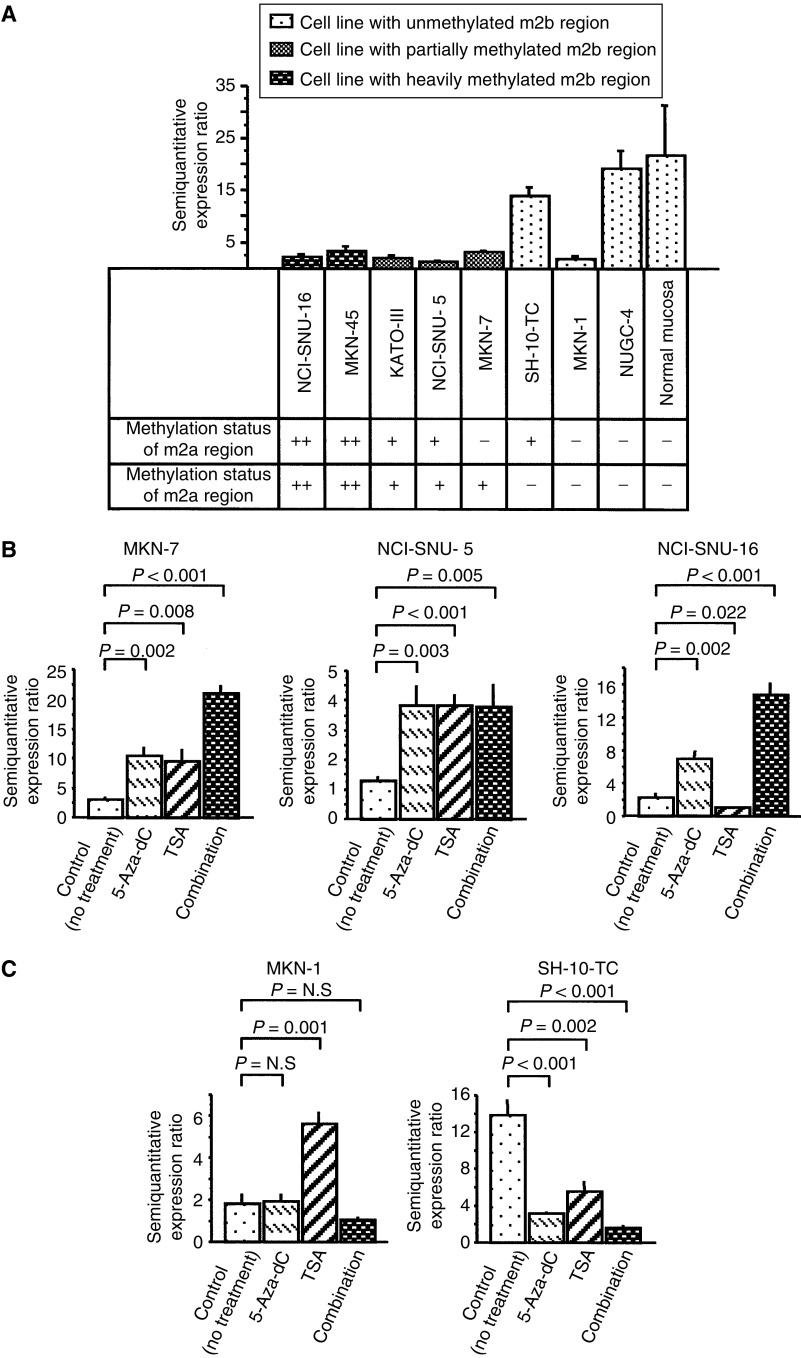
*hDAB2IP* mRNA expression using semiquantitative real-time RT–PCR. Triplicate sets of flasks before and after drug treatment were harvested and tested for gene expression using semiquantitative real-time PCR. Data shown are mean values±s.d., *n*=3. (**A**) The relative ratio of mRNA expression in each cell line. The relative expression ratio was remarkably repressed in m2b-methylated cell lines compared with m2b-unmethylated ones except MKN-1. Following symbols of *two plus* and *minus* means positive and negative for methylation status by MSP, respectively. *One plus* means positive for both the methylated and unmethylated forms by MSP. (**B**) Effect of 5-Aza-CdR, TSA and combination on mRNA expression in m2b-methylated cell lines (MKN-7, NCI-SNU-5 and NCI-SNU-16). *hDAB2IP* expression of MKN-7 was upregulated 3.4-fold by 5-Aza-CdR, 3.1-fold by TSA and additively 6.9-fold by combination. In NCI-SNU-5, *hDAB2IP* expression was also upregulated 3.0-fold by 5-Aza-CdR and 3.0-fold by TSA, whereas TSA failed to make synergistic or additive effect for this cell line. In NCI-SNU-16, the expression was also upregulated 3.1-fold by 5-Aza-CdR, but TSA alone failed to make a positive effect, operating synergically to 6.6-fold on the expression with a combination of 5-Aza-CdR. (**C**) Effect of 5-Aza-CdR, TSA and combination on mRNA expression in m2b-unmethylated cell lines (MKN1 and SH-10-TC). In MKN-1, the downregulated expression was restored 3.1-fold by TSA alone. In SH-10-TC, the expression was decreased after any drug treatment, which may be caused by a cytotoxic effect from drug exposure.

We examined the expression status of *hDAB2IP* by semiquantitative real-time RT–PCR in eight cell lines, consisting of three showing heavy methylation in both the m2a and m2b regions, one showing an m2a-dominant methylation pattern, two showing an m2b-dominant methylation pattern, and two showing no methylation of the *hDAB2IP* gene. The semiquantitatively estimated mRNA expression values in each cell line are shown in Figure 2A. The values in the two cell lines showing no methylation of the gene were as follows; the mean *hDAB2IP* expression ratio in NUGC-4 was very high at 19.2, as expected, but the ratio in MKN-1 was quite unexpectedly low, with a value of 1.81. This transcriptional downregulation of *hDAB2IP* in MKN-1 was attributed to treatment with a histone deacetylase inhibitor, as described below. As compared with the value in NUGC-4, the expression ratios in all of the cell lines showing methylation of the *hDAB2IP* gene, with the exception of SH-10-TC, were significantly lower, with the mean ratios in NCI-SNU-16, MKN-45, KATO-III, NCI-SNU-5 and MKN-7 being 2.24, 3.23, 2.08, 1.28, and 3.03 respectively; the expression ratio in SH-10-TC was only 13.8. SH-10-TC showed a moderate methylation status with the m2a-dominant pattern, but its m2b region was almost completely unmethylated, as described above. On the other hand, in MKN-7 and NCI-SNU-5, in which the expression ratios indicated significant downregulation of *hDAB2IP*, the m2a-methylation rates were equal to that in SH-10-TC, but the m2b regions were additionally methylated (Figure 1B). These results indicate that the expression of *hDAB2IP* tended to be reduced depending on the methylation status in the m2b region, which is just adjacent to the transcriptional-start site, and suggest that the m2b promoter region might be the methylation-mediated key regulator of the expression of *hDAB2IP* in gastric cancer. In the five nonmalignant gastric mucosal specimens examined, the mean expression ratio of *hDAB2IP* was 21.6±9.7, indicative of normal expression.

### MSP assay and aberrant methylation of *hDAB2IP* in gastrointestinal tumours

**Figure 3:**
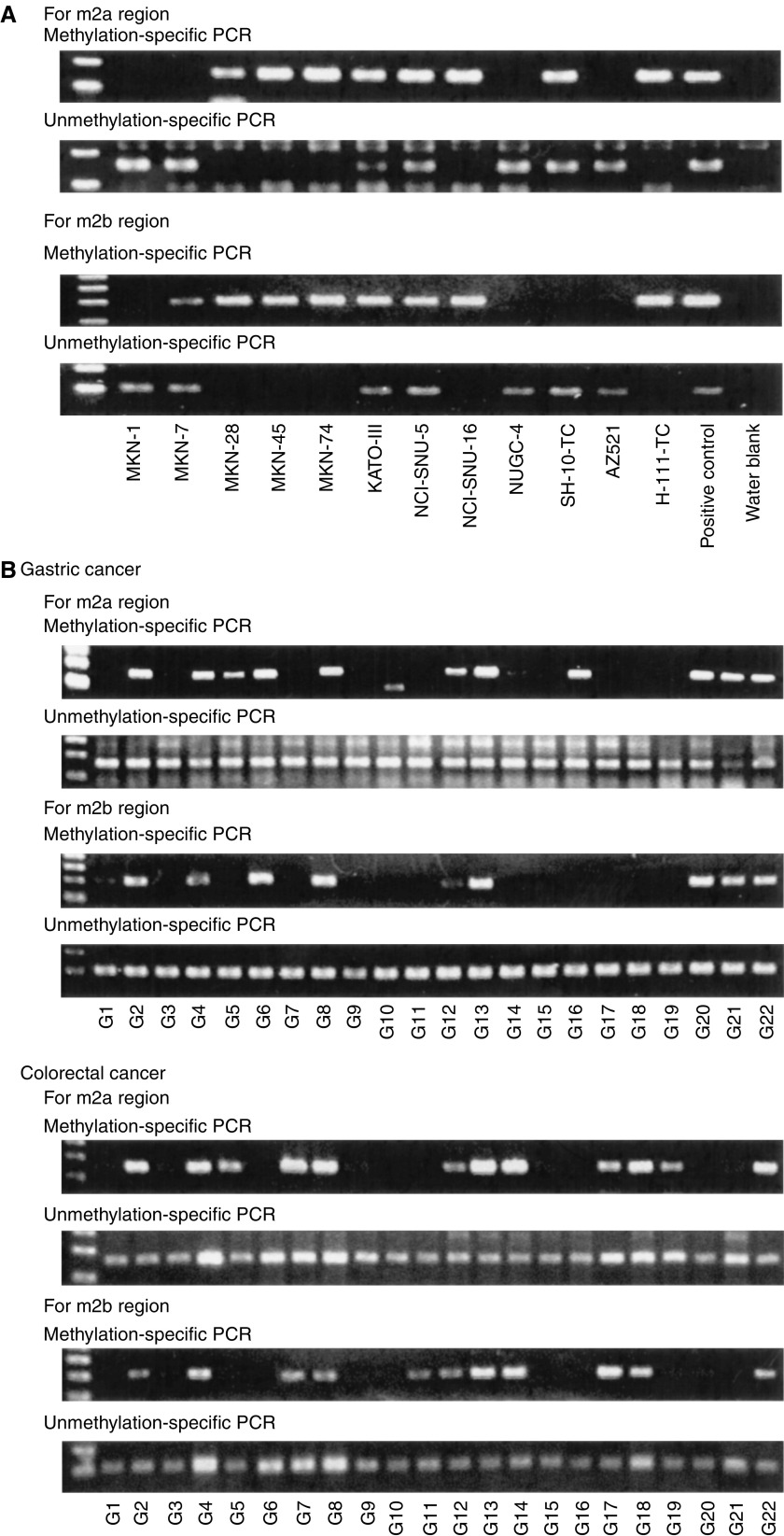
Representative examples of MSP in gastrointestinal tumours. (**A**) gastric cancer cell lines, (**B**) primary tumours. The unmethylated form of *hDAB2IP* was always found in primary tumours that had been grossly dissected and thus had at least some contamination with normal cells. Positive controls include peripheral blood lymphocytes from healthy individuals for the unmethylated form and normal lymphocyte DNA methylated by treatment with Sss I DNA methyltransferase for the methylated form.

We determined the forward and reverse primers for MSP of each of the m2a and m2b regions (Figure 1B). The CpG sites in these regions were almost consistently methylated in the expression-reduced cell lines (Figure 1B). Three and four cytosines were included in the forward and reverse primers, respectively, for the m2a region producing a 163-bp amplicon, and five and three cytosines were included in the forward and reverse primers, respectively, for the m2b region producing a 209-bp amplicon (Figure 1B). Methylation-specific PCR assay using these primers was performed to examine the methylation status of *hDAB2IP* in the gastric cancer cell lines and in primary gastric and colorectal cancer tumours. Representative examples of the assay are illustrated in Figure 3A and B and the results are summarised in Table 2 . Aberrant methylation of the m2a and m2b regions was found in 16 of 35 (46%) and 15 of 35 (43%) primary gastric cancer specimens, respectively, and in 29 of 60 (48%) and 28 of 60 (47%) primary colorectal cancer specimens, respectively. In the case of the gastric cancer cell lines, methylation of the gene was detected in eight of 12 cell lines (67%) for each of the m2a and m2b regions. The unmethylated form of *hDAB2IP* was always found in primary tumour samples because these had been grossly dissected and thus showed some contamination with normal cells. In contrast, the cancer cell lines consisted of a pure population of tumour cells, and eight of the 12 cell lines (67%) had either methylated or unmethylated *hDAB2IP* alleles for both m2a and m2b regions. Four of the 12 cell lines (33%) showed both methylated and unmethylated forms for the m2a or the m2b region, and were regarded as showing partial methylation of the gene, and the details of the CpG methylation status in these cell lines are shown in Figure 1B. A total of 36 (38%) primary gastric and colorectal cancer specimens and seven (58%) cell lines showed aberrant methylation in both the m2a and m2b regions. On the other hand, 16 (17%) primary gastric and colorectal cancer specimens and two (17%) cell lines showed methylation of either the m2a or the m2b region. There were no significant differences in the methylation rate between the cancer cell lines and primary tumours in either the m2a or the m2b region. In addition, seven (12%) and 11 (19%) of 59 gastrointestinal normal mucosal specimens showed methylation in the m2a and m2b regions, respectively. Aberrant methylation was not present in either of the two regions in samples obtained from five peripheral blood lymphocytes.

### 5-Aza-CdR and TSA treatment for gastric cancer cell lines

To confirm the involvement of DNA methylation in *hDAB2IP* silencing, we treated five cell lines with 5-Aza-CdR. *hDAB2IP* expression was upregulated following the 5-Aza-CdR treatment in the m2b-methylated cell lines. In contrast, no such upregulation following 5-Aza-CdR treatment was observed in the m2b-nonmethylated cell lines. This finding indicates that methylation in the m2b region plays an important role in regulating the gene silencing. Furthermore, we also treated cell lines with TSA, either singly or in combination with 5-Aza-CdR. Expression of *hDAB2IP* in MKN-7 was upregulated 3.1-fold by TSA treatment and by 6.9-fold by following combined treatment with TSA and 5-Aza-CdR. In NCI-SNU-5, while *hDAB2IP* expression was upregulated 3.0-fold by treatment with TSA alone, combined treatment with TSA and 5-Aza-CdR failed to elicit a synergistic or additive effect. In NCI-SNU-16, while treatment with TSA alone failed to have any positive effect, combined treatment with 5-Aza-CdR elicited a positive effect on *hDAB2IP* expression.

On the other hand, in SH-10-TC, an m2b-nonmethylated cell line, treatment with the aforementioned drugs neither upregulated nor downregulated the expression of *hDAB2IP* (0.1–0.4-fold), because the number of SH-10-TC cells in culture was decreased by treatment with 5-Aza-CdR, TSA or a combination of 5-Aza-CdR and TSA (data not shown). This effect may be attributable to the cytotoxic effect of 5-Aza-CdR and TSA. Interestingly, in MKN-1, which showed no methylation in either the m2a or the m2b region, the downregulated expression of *hDAB2IP* could be restored by TSA treatment alone. This result suggests that the expression of the gene might be downregulated under the control of histone deacetylation in this cell line alone. These results suggest that both DNA methylation and histone deacetylation may act cooperatively to silence *hDAB2IP* expression in some gastric cancer cell lines, and that the methylation status of the m2b region may act as the major gene silencing mechanism in most cell lines.

### *hDAB2IP* methylation and clinicopathological correlation

We examined the relationship between the methylation status in the tumour specimens and clinicopathological factors, including sex, age, tumour size, extent of tumour invasion, lymph node status, distant metastasis, TNM stage, histology and the tumour site in cases with gastrointestinal cancers. In cases of gastric cancer, while both the m2a- and m2b-methylation status were significantly associated with the tumour site, the m2b-methylation status showed a stronger correlation than the m2a-methylation status (m2a, *P*=0.0172; m2b, *P*=0.0064; Table 3 ). In cases of colorectal cancer, m2b methylation tended to be associated with cancer arising from the left side of the colon. (*P*=0.0926; Table 4 ). *hDAB2IP* methylation was often observed in tumours arising from the lower-third of the stomach and distal aspect of the colorectum. There were no significant differences between methylation-positive and methylation-negative groups with respect to the other factors examined in this study. Although accumulation of methylation appears to be age-related in a number of cancer-related gene*s*, both the m2a- and m2b-methylation status were not significantly associated with the age of patients (gastric cancer: m2a, *P*=0.9369; m2b, *P*=0.9788; colorectal cancer: m2a, *P*=0.8484; m2b, *P*=0.5709; Table 5 ).

## DISCUSSION

To determine the methylation status of the 5′ CpG islands of *hDAB2IP* in gastrointestinal tumours, we first performed bisulphite genomic DNA sequencing in several gastric cancer cell lines. Based on the sequence data, methylation-specific primers were designed to detect sensitively each methylated or unmethylated allele (Toyooka *et al*, 2002b, 2003). These primers have also been shown to be applicable to lung and breast cancer cell lines (Dote *et al*, 2004). Using the MSP primers designed thus, we found frequent methylation of the *hDAB2IP* gene in primary gastrointestinal tumours, as well as in gastrointestinal tumour cell lines. The higher frequency of methylation in the cell lines as compared with that in the primary tumours might be explained by the additional changes that the cells may acquire in culture, or by the tumour cell lines being derived from more aggressive tumours and therefore showing more malignant changes (Zochbauer-Muller *et al*, 2001).

We previously reported that the m2a region appeared to be the key regulatory region for *hDAB2IP* expression in prostate cancer (Chen *et al*, 2003) and that methylation of both the m2a and m2b regions occurred in breast cancer cell lines (Dote *et al*, 2004). However, in contrast to the observation in prostate and breast cancer, this study demonstrated that the downregulation of *hDAB2IP* expression correlated more with the methylation status of not the m2a region, but of the m2b region, which is located just adjacent to exon Ia in gastric cancer cell lines (Figure 2A). Considering the close proximity of the m2b region to the transcriptional-start site of the *hDAB2IP* gene, it stands to reason that the m2b region might act as the regulatory promoter in gastric cancer cells.

We demonstrated in this study that the expression of *hDAB2IP* was highly suppressed in five m2b-methylated cell lines as compared with that in two m2b-nonmethylated cell lines. This result suggests that DNA methylation of the regulatory promoter region may be critical for transcriptional regulation of the *hDAB2IP* gene and play an important role in *hDAB2IP* inactivation. These results are consistent with the finding of restoration of *hDAB2IP* expression in methylated cell lines after 5-Aza-CdR treatment.

Recent data suggest that hypermethylated DNA interacts with several methyl-CpG-binding proteins, and that the interaction facilitates the assembly of a repressive complex containing histone deacetylase and formation of an inactive chromatin that leads to gene silencing (Nan *et al*, 1998; Wade *et al*, 1999). Therefore, we examined the effect of the histone deacetylase inhibitor, TSA, as well as that of 5-Aza-CdR on the methylated cell lines, to distinguish the effect of histone deacetylation on the downregulated gene expression of *hDAB2IP*. In MKN-7, the expression was upregulated by both 5-Aza-CdR and TSA treatment, and combined treatment with both agents caused synergistic upregulation, with the extent of upregulation in this case being greater than that with either agent alone. In addition, in some other methylated cells, TSA enhanced *hDAB2IP* gene expression. These results indicate that in some cell lines showing gene methylation, there may be a synergistic and positive interaction between DNA methylation and histone deacetylation for transcriptional regulation, although this does not seem to be a common and major mechanism in gastric cancer cells. However, the number of examined cells was too limited to arrive at any definitive conclusion on the effect of TSA on *hDAB2IP* expression. Considering this effect of deacetylation on gene expression, it was reported that the expression of some methylation-repressed genes can be reactivated by TSA treatment (Yang *et al*, 2000) and that demethylation-induced gene re-expression could be potentiated by TSA (Cameron *et al*, 1999). In addition, Suzuki *et al* (2002) analysed the expression of over 10 000 genes by microarray analysis, and found that the expression of 74 genes was upregulated following treatment with 5-Aza-CdR or TSA. They also showed that transcriptional silencing is mediated by both methylation and histone deacetylation, although the effect of methylation was dominant for gene silencing.

*hDAB2IP* methylation was found to be present at similar frequencies in both early and advanced carcinomas, suggesting that the methylation actually occurs at an early stage of carcinogenesis in gastrointestinal tumours. Furthermore, the result that *hDAB2IP* methylation was found in nonmalignant mucosa suggested that *hDAB2IP* methylation might play a causative role in carcinogenesis. Interestingly, we found a highly significant association between the tumour location in the stomach and methylation of the m2b region, which has been supposed to be the key regulatory sequence, as described above. Cancers showing methylation of the m2b region showed a significant tendency towards being preferentially located in the lower-third of the stomach. Similar results were also described for *p16*, *hMLH1* and *TSLC1* methylation in gastric cancer (Schneider *et al*, 2000; Honda *et al*, 2002; Sakata *et al*, 2002). Therefore, methylation of several tumour-suppressor and tumour-related genes appears to be an important pathogenetic mechanism of gastric cancers arising from the antrum. Because intestinal metaplasia commonly arises in the antrum and then extends towards the body of the stomach, intestinal metaplasia may predispose to promoter region methylation in these genes (Sato *et al*, 2001). In addition, a marked increase in the frequency of methylated genes from nonmetaplastic mucosa to intestinal metaplasia has been reported (Kang *et al*, 2001). In sporadic colorectal cancers, it has recently been shown that CpG island methylation is associated with a right-sided location of the tumour (Hawkins *et al*, 2002). However, we also found an inverse association between m2b-region methylation and a right-sided location of the tumour, although this did not reach statistical significance.

In conclusion, our results demonstrate that aberrant methylation drives gene downregulation, and supports the contention that the methylation-mediated transcriptional silencing of the *hDAB2IP* gene may be a critical event in tumorigenesis of gastrointestinal tumours, especially those arising from the antrum of the stomach in association with intestinal metaplasia.

## Figures and Tables

**Table 1:** Clinical characteristics of studied gastric and colorectal cancer patients

**Variables**	**No. of informative gastric cancer cases (%)**	**No. of colorectal cancer informative cases (%)**
*Sex*		
Male	21 (60)	37 (62)
Female	14 (40)	23 (38)
		
*Tumour invasion*		
T 1	6 (17)	4 (7)
T 2	14 (40)	4 (7)
T 3	11 (31)	45 (75)
T 4	4 (12)	6 (10)
		
*Nodal status*		
N 0	14 (40)	25 (42)
N 1	14 (40)	18 (30)
N 2	6 (17)	17 (28)
N 3	1 (3)	—
		
*Distant metastasis*		
M 0	26 (74)	37 (62)
M 1	9 (26)	23 (38)
		
*TNM staging*		
Stage I	10 (29)	7 (12)
Stage II	8 (23)	13 (22)
Stage III	6 (17)	16 (27)
Stage IV	11 (31)	23 (38)

**Table 2:** Aberrant promoter methylation of *hDAB2IP* in samples

**Samples**	**No. of *hDAB2IP* m2a methylated samples (%)**	**No. of *hDAB2IP* m2b methylated samples (%)**	**No. of both m2a and m2b methylated samples (%)**
*Gastric cancers*			
Cell lines (*n*=12)	8 (67)	8 (67)	7 (58)
Primary tumours (*n*=35)	16 (46)	15 (43)	13 (37)
			
*Colorectal cancers*			
Primary tumours (*n*=60)	29 (48)	28 (47)	23 (38)
			
*Nonmalignant specimens (n=64)*			
Normal gastric mucosa (*n*=35)	2 (6)	7 (20)	2 (6)
Normal colorectal mucosa (*n*=24)	5 (21)	4 (17)	3 (13)
Peripheral lymphocytes (*n*=5)	0 (0)	0 (0)	0 (0)

**Table 3:** Clinicopathologic features and *hDAB2IP* promoter methylation in gastric cancer

**Clinicopathologic features**	**Variable^a^**	**Frequency of *hDAB2IP* m2a methylation (%)**	***P*-value^b^**	**Frequency of *hDAB2IP* m2b methylation (%)**	***P*-value^b^**
Sex	Male (*n*=21)	8 (38)	0.27	9 (43)	>0.99
	Female (*n*=14)	8 (57)		6 (43)	
Tumour invasion	T1, 2 (*n*=20)	9 (45)	0.92	9 (45)	0.77
	T3, 4 (*n*=15)	7 (47)		6 (40)	
Nodal metastasis	N0 (*n*=14)	7 (50)	0.68	7 (50)	0.49
	N1, 2 (*n*=21)	9 (43)		8 (38)	
Metastasis	M0 (*n*=26)	12 (46)	0.93	11 (42)	0.91
	M1 (*n*=9)	4 (44)		4 (44)	
TNM stage	1, 2 (*n*=18)	8 (44)	0.88	8 (44)	0.85
	3, 4 (*n*=17)	8 (47)		7 (41)	
Histology	Intestinal (*n*=17)	8 (47)	0.88	6 (35)	0.38
	Diffuse (*n*=18)	8 (44)		9 (50)	
Tumour site	U (*n*=7)	2 (22)	0.017	1 (14)	0.0064
	M (*n*=13)	3 (23)		3 (23)	
	L (*n*=15)	11 (73)		11 (73)	

a U=upper site; M=middle site; L=lower site.

b Statistical significance was determined using *χ*^2^ test.

**Table 4:** Clinicopathologic features and *hDAB2IP* promoter methylation in colorectal cancer

**Clinicopathologic features**	**Variable^a^**	**Frequency of *hDAB2IP* m2a methylation (%)**	***P*-value^b^**	**Frequency of *hDAB2IP* m2b methylation (%)**	***P*-value^b^**
Sex	Male (*n*=37)	18 (49)	0.95	15 (41)	0.23
	Female (*n*=23)	11 (48)		13 (57)	
Tumour invasion	T1, 2 (*n*=9)	4 (44)	0.80	5 (56)	0.56
	T3, 4 (*n*=51)	25 (49)		23 (45)	
Nodal metastasis	N0 (*n*=25)	9 (36)	0.11	11 (44)	0.73
	N1, 2 (*n*=35)	20 (57)		17 (49)	
Metastasis	M0 (*n*=53)	26 (49)	0.76	25 (47)	0.83
	M1 (*n*=7)	3 (43)		3 (43)	
TNM stage	1, 2 (*n*=21)	7 (33)	0.088	9 (43)	0.66
	3, 4 (*n*=39)	22 (56)		19 (49)	
Histological grade	G1 (*n*=13)	7 (54)	0.65	6 (46)	0.97
	G2, 3, 4 (*n*=47)	22 (47)		22 (47)	
Tumour site	C, A, T (*n*=12)	4 (33)	0.25	3 (25)	0.093
	D, S, R (*n*=48)	25 (52)		25 (52)	

a C=cecum; A=ascending colon; T=transverse colon; D=descending colon; S=sigmoid colon; R=rectum.

b Statistical significance was determined using *χ*^2^ test.

**Table 5:** Association between *hDAB2IP* promoter methylation status and age in gastrointestinal cancer

**Methylation status**	**Age^a^**	***P*-value^b^**
*Gastric cancer*		
m2a-methylation (*n*=15)	66 (40–89)	0.9369
m2a-unmethylation (*n*=20)	66 (36–85)	
m2b-methylation (*n*=16)	66 (40–89)	0.9788
m2b-unmethylation (*n*=19)	66 (36–85)	
		
*Colorectal cancer*		
m2a-methylation (*n*=29)	66 (51–85)	0.8484
m2a-unmethylation (*n*=31)	65 (26–86)	
m2b-methylation (*n*=28)	66 (47–82)	0.5709
m2b-unmethylation (*n*=32)	65 (26–86)	

a Mean age (range) in years.

b Statistical significance was determined using Mann–Whitney *U*-test.
